# α‐Synuclein Radiotracer Development and *In Vivo* Imaging: Recent Advancements and New Perspectives

**DOI:** 10.1002/mds.28984

**Published:** 2022-03-15

**Authors:** Obada M. Alzghool, Guus van Dongen, Elsmarieke van de Giessen, Linda Schoonmade, Wissam Beaino

**Affiliations:** ^1^ Department of Radiology and Nuclear Medicine, Tracer Center Amsterdam Amsterdam UMC, Vrije Universiteit Amsterdam The Netherlands; ^2^ Turku PET Centre University of Turku Turku Finland; ^3^ Medical Library Vrije Universiteit Amsterdam Amsterdam The Netherlands

**Keywords:** α‐synuclein, α‐synucleinopathies, Parkinson's disease, positron emission tomography, radiotracers

## Abstract

α‐Synucleinopathies including idiopathic Parkinson's disease, dementia with Lewy bodies and multiple systems atrophy share overlapping symptoms and pathological hallmarks. Selective neurodegeneration and Lewy pathology are the main hallmarks of α‐synucleinopathies. Currently, there is no imaging biomarker suitable for a definitive early diagnosis of α‐synucleinopathies. Although dopaminergic deficits detected with single‐photon emission computed tomography (SPECT) and positron emission tomography (PET) radiotracers can support clinical diagnosis by confirming the presence of dopaminergic neurodegeneration, dopaminergic imaging cannot visualize the preceding disease process, nor distinguish α‐synucleinopathies from tauopathies with dopaminergic neurodegeneration, especially at early symptomatic disease stage when clinical presentation is often overlapping. Aggregated α‐synuclein (αSyn) could be a suitable imaging biomarker in α‐synucleinopathies, because αSyn aggregation and therefore, Lewy pathology is evidently an early driver of α‐synucleinopathies pathogenesis. Additionally, several antibodies and small molecule compounds targeting aggregated αSyn are in development for therapy. However, there is no way to directly measure if or how much they lower the levels of aggregated αSyn in the brain. There is clearly a paramount diagnostic and therapeutic unmet medical need. To date, aggregated αSyn and Lewy pathology inclusion bodies cannot be assessed ante‐mortem with SPECT or PET imaging because of the suboptimal binding characteristics and/or physicochemical properties of current radiotracers. The aim of this narrative review is to highlight the suitability of aggregated αSyn as an imaging biomarker in α‐synucleinopathies, the current limitations with and lessons learned from αSyn radiotracer development, and finally to propose antibody‐based ligands for imaging αSyn aggregates as a complementary tool rather than an alternative to small molecule ligands. © 2022 The Authors. *Movement Disorders* published by Wiley Periodicals LLC on behalf of International Parkinson Movement Disorder Society.

## Graphical abstract

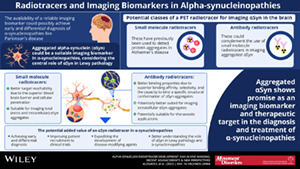

May Infographic : Alpha‐Synuclein Radiotracer Development and In Vivo Imaging: Recent Advancements and New Perspectives

α‐Synucleinopathies form a subset of neurological disorders that include idiopathic Parkinson's disease (PD), dementia with Lewy bodies (DLB), multiple systems atrophy (MSA), and some rare disorders, such as pure autonomic failure.^1^ These disorders share unclear etiology, overlapping symptoms and pathological hallmarks.^2^, ^3^, ^4^, ^5^, ^6^, ^7^, ^8^ Neurological disorders are the leading cause of disability in the world, the fastest growing of which is PD surpassing even Alzheimer's disease (AD).^9^ PD is also the most common type of α‐synucleinopathies. The Global Burden of Disease Study in 2016 estimated that 6.1 million individuals had PD and that number is rising exponentially.^10^ Aging is the greatest risk factor for PD and other α‐synucleinopathies. Genetic factors also affect disease risk, onset, and progression. There are variants in more than 20 genes reported to cause PD.^11^ Several missense mutations in *SNCA*, the α‐synuclein (αSyn) encoding gene, are proven to cause parkinsonism.^12^, ^13^, ^14^, ^15^, ^16^, ^17^, ^18^ Gene multiplication is another abnormality associated with *SNCA* where the extra gene copies may cause an increased expression of αSyn and an increased tendency to self‐aggregation and malfunction.^19^, ^20^, ^21^

αSyn is a ubiquitous protein in the central nervous system accounting for up to 1% of the total cytosol proteins.^22^, ^23^ αSyn is predominantly expressed in the brain, and concentrated at the pre‐synaptic nerve terminals.^24^ The precise function of αSyn at physiological conditions remains unclear; however studies using cellular and animal models show that αSyn contributes to synaptic vesicles trafficking and neurotransmitter release.^25^ In vitro aggregation studies revealed that wild type and mutated variants of recombinant human αSyn monomers aggregate to form oligomers, which further aggregate into fibrils, a process associated with structural transition from random coil to β‐sheet.^26^

The main hallmarks of α‐synucleinopathies are selective dopaminergic neurodegeneration and Lewy pathology being the process of αSyn aggregation into inclusion bodies.^27^, ^28^, ^29^ Lewy pathology plays a central role in the pathogenesis of α‐synucleinopathies as one of the main drivers of neurodegeneration. This happens via the ongoing process of αSyn aggregation, which disrupts cellular functions, and induces mitochondrial damage and synaptic dysfunction.^30^ Lewy pathology develops in different anatomic patterns and cell types in different α‐synucleinopathies. Neuropathological studies show that in PD and DLB αSyn accumulates inside the neurons as Lewy bodies (LBs) and Lewy neurites (LNs),^31^, ^32^ whereas in MSA, αSyn accumulates inside oligodendrocytes as glial cytoplasmic inclusions (GCIs) and intraneuronal inclusions (Fig. 1A).^33^, ^34^ In vitro and in vivo studies using primary neurons, rodents and patient brain tissue have demonstrated that GCI αSyn and LB αSyn strains are formed based on the different intracellular environments in oligodendrocytes and neurons, respectively, and that these strains possess distinct structural and biological properties. GCI αSyn aggregates are more structurally compact compared to LB αSyn. In addition, GCI αSyn demonstrate higher potency in inducing Lewy pathology and different pattern of transmission.^35^, ^36^

**FIG 1:**
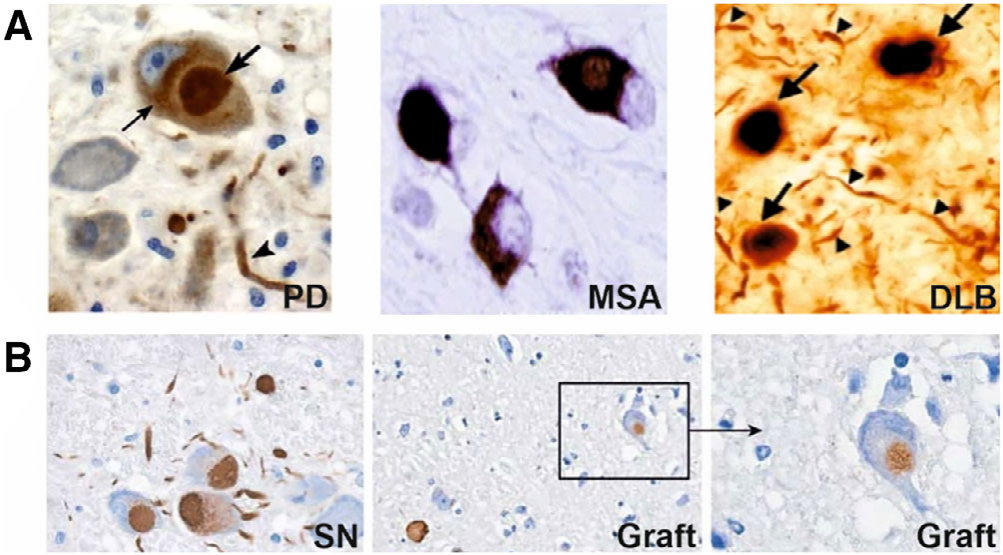
Lewy pathology in different α‐synucleinopathies shown by αSyn immunohistochemical staining in Parkinson's disease (PD) and multiple system atrophy (MSA) using paraffin‐embedded brain tissue sections, and in dementia with Lewy bodies (DLB) using floating brain tissue sections. (**A**) Representative examples of Lewy bodies (bold arrow), Lewy neuritis (arrowhead) and αSyn aggregates (light arrow) in PD and DLB and glial cytoplasmic inclusions in MSA.^32^, ^34^ (**B**) Lewy bodies and Lewy neurites in neurons of the substantia nigra (left) propagate to grafted neurons (middle and right) in PD.^100^

Lewy pathology spreading in PD brain tends to follow a consistent pattern. LBs and LNs first appear in the medulla oblongata and olfactory bulbs, then cover the pontine tegmentum, midbrain, limbic brain regions, and eventually extend into the neocortex.^37^ In DLB, Lewy pathology is widely spread and depending on the distribution, pathology is classified as brainstem, limbic, or neocortical‐predominant.^38^ It should be noted that DLB and PD are increasingly considered a disease continuum based on similar pathology, although in DLB and the intermediate form Parkinson's disease dementia (PDD), dementia is at the foreground of the clinical presentation.^39^ Lewy pathology in MSA spreads from the basal ganglia, brainstem, and cortex into the cervical spinal cord and thalamus, then to the hippocampus and amygdala, and eventually into the occipital neocortex.^40^ The unified staging system for Lewy body disorders^41^ and the staging/typing of Lewy body‐related α‐synuclein pathology^42^ are clinically used assessment protocols of Lewy pathology progression in different α‐synucleinopathies.

## Diagnosis and Therapy of α‐Synucleinopathies

Diagnosis of α‐synucleinopathies is performed according to clinical criteria. For example, clinical diagnosis of PD is based on the presence of bradykinesia in combination with at least resting tremor or rigidity and at least two supportive criteria (eg, beneficial response to dopaminergic therapy) without exclusion criteria (eg, cerebellar abnormalities or normal presynaptic dopaminergic imaging).^43^ DLB is clinically diagnosed as progressive dementia accompanied with fluctuating cognition, visual hallucinations, parkinsonism, and REM sleep behavior disorder.^44^ MSA is clinically diagnosed as autonomic dysfunction and either poorly levodopa‐responsive parkinsonism or a cerebellar syndrome.^45^

**FIG 2:**
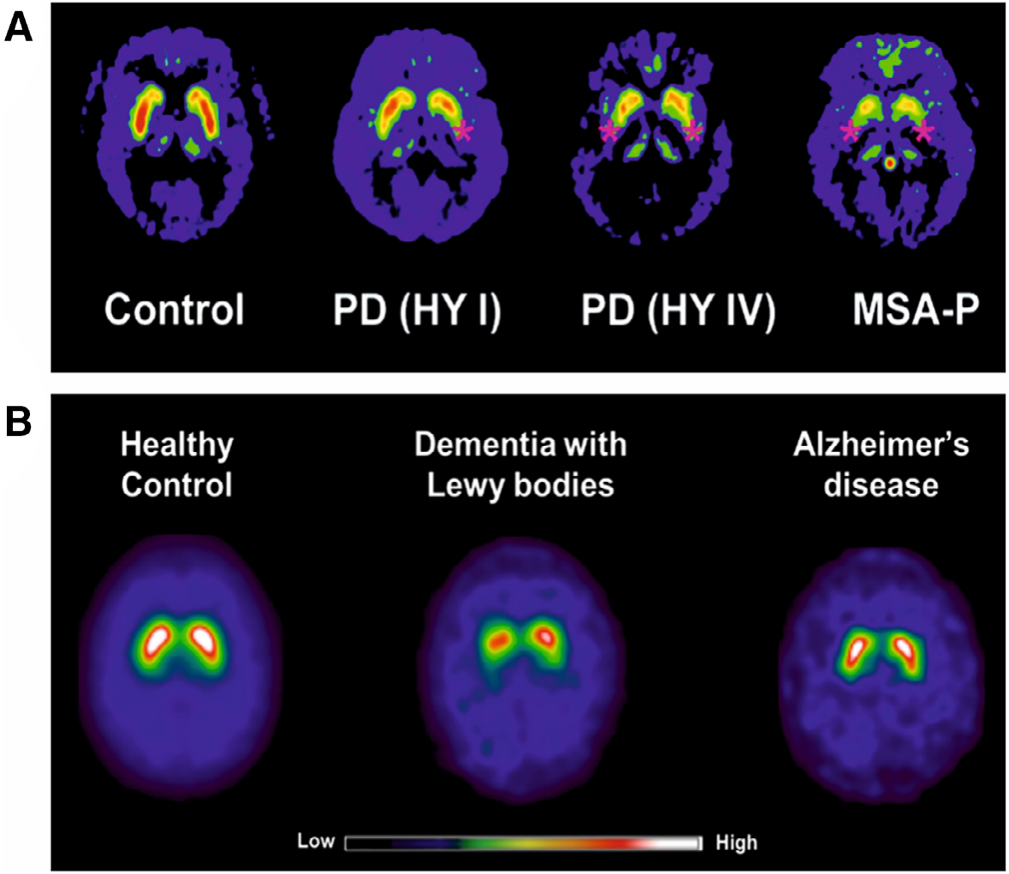
Representative examples of dopamine transporter (DAT) imaging in α‐synucleinopathies. (**A**) [^18^F]FDOPA uptake is reduced (red stars) in the putamen in Parkinson's disease early (PD HY I) and advanced (PD HY IV) stages, and multiple system atrophy with parkinsonian symptoms (MSA‐P) compared to control.^104^ (**B**) [^123^I]FP‐CIT show reduced DAT uptake in dementia with Lewy bodies, whereas DAT uptake is normal in healthy controls and Alzheimer's disease.^48^

Single‐photon emission computed tomography (SPECT) and positron emission tomography (PET) imaging could support the clinical diagnosis of α‐synucleinopathies. Pre‐synaptic dopaminergic imaging with [^123^I]FP‐CIT SPECT and [^18^F]FDOPA PET is used to distinguish neurodegenerative parkinsonisms (PD, MSA, progressive supranuclear palsy [PSP] and cortico‐basal degeneration [CBD]) from non‐neurodegenerative parkinsonisms (eg, essential tremor, vascular or medication induced parkinsonism) with high sensitivity (98%) and specificity (98%).^46^ Pre‐synaptic dopaminergic imaging is also clinically useful to distinguish DLB from AD (Fig. 2).^47^, ^48^ It can even be used to assess dopaminergic neurodegeneration in prodromal symptoms such as REM sleep behavior disorder^49^ and hyposmia.^50^ Although dopaminergic deficits detected with SPECT and PET radiotracers can support clinical diagnosis by confirming the presence of dopaminergic neurodegeneration, dopaminergic imaging cannot visualize the preceding disease process. Approximately 50% of nigral dopaminergic neurons are already degenerated at motor symptom manifestation and current clinical diagnosis.^51^ Diagnosing α‐synucleinopathies is ideally performed at an earlier stage when neurodegeneration is still limited. This would provide a window of opportunity for therapeutic intervention to prevent, slow down, or halt the disease progression. Another clinical challenge is the differential diagnosis of atypical parkinsonisms, especially at early symptomatic disease stage when clinical presentation is often overlapping. Presynaptic dopaminergic imaging cannot reliably distinguish PD from the atypical parkinsonisms MSA, PSP, and CBD. [^18^F]FDG brain PET imaging can be used to aid the differential diagnosis of atypical parkinsonisms, but its reading requires training and semi‐quantitative analysis software, and its value in the pre‐symptomatic phase is probably limited.^52^ A good imaging biomarker to distinguish α‐synucleinopathies (PD, MSA) from non‐α‐synucleinopathies (in particular the tauopathies PSP and CBD) could be clinically useful.^53^

Currently, there is no reliable imaging biomarker for an early and definitive ante‐mortem diagnosis of α‐synucleinopathies. Lewy pathology is an early driver of α‐synucleinopathies pathogenesis. There is evidence for an association between Lewy pathology and clinical symptoms severity,^41^, ^54^, ^55^ neuronal dysfunction,^56^, ^57^ and decreased nigral neuronal density in early Braak stages.^58^ Aggregated αSyn could be a suitable imaging biomarker in α‐synucleinopathies. However, currently, aggregated αSyn and Lewy pathology inclusion bodies cannot be assessed ante‐mortem with SPECT or PET radiotracers. An αSyn specific radiotracer capable for early diagnosis of α‐synucleinopathies, potentially aiding differential diagnosis, is highly desired.

Additionally, as might be concluded from the information above, aggregated αSyn could be a suitable target for therapy in α‐synucleinopathies considering its key role in Lewy pathology. There are currently no registered disease‐modifying agents available for the treatment of α‐synucleinopathies, current treatments are only acting on symptoms.^59^ Several biological^60^, ^61^, ^62^, ^63^, ^64^, ^65^, ^66^, ^67^, ^68^ and small molecule compounds^69^, ^70^, ^71^ targeting αSyn aggregates are in clinical and pre‐clinical development. However, currently there is no way to directly measure if or how much they lower the levels of aggregated αSyn in the brain. Ideally, the same compound targeting aggregated αSyn can be used for therapy where it exerts a desired clinical effect and as a radiotracer for diagnosis and therapy monitoring where it confirms the specific target engagement and demonstrates the clinical benefit. The availability of αSyn radiotracer for monitoring αSyn load, target engagement, disease progression, and therapy response would greatly benefit the development process. In a comparable way, the availability of PET radiotracers for amyloid pathology in AD facilitated the clinical development of aducanumab in terms of clinical trials subject recruitment and efficacy evaluation,^72^, ^73^, ^74^ despite the controversial and yet to be established efficacy and clinical benefit of aducanumab. This example in the field of neurodegeneration demonstrates the potential of an αSyn radiotracer facilitating targeted therapy development in α‐synucleinopathies.

## Strategies for αSyn Radiotracer Development

Recommendations in the αSyn imaging field highlight the pressing need for SPECT and PET radiotracers to aid the diagnosis and treatment in α‐synucleinopathies. The Michael J. Fox Foundation established the α‐Synuclein Imaging Consortium in 2011, launched a $2 million prize for the development of a selective αSyn PET radiotracer in 2016, and in 2019 announced the $10 million Ken Griffin Alpha‐synuclein Imaging Competition. Despite these efforts, to date there is no radiotracer available for imaging αSyn. Several small‐molecule ligands have been developed and tested for in vivo imaging and detection of aggregated αSyn and Lewy pathology inclusion bodies (LBs/LNs/GCIs) in α‐synucleinopathies. This part of the review covers the used strategies for αSyn ligand development and findings from ligands that advanced to pre‐clinical and clinical evaluation. More extensive and radiochemical‐oriented description of all developed and tested probes targeting aggregated αSyn has been reviewed elsewhere.^75^, ^76^, ^77^, ^78^

## Repurposed Radiotracers from AD

**FIG 3:**
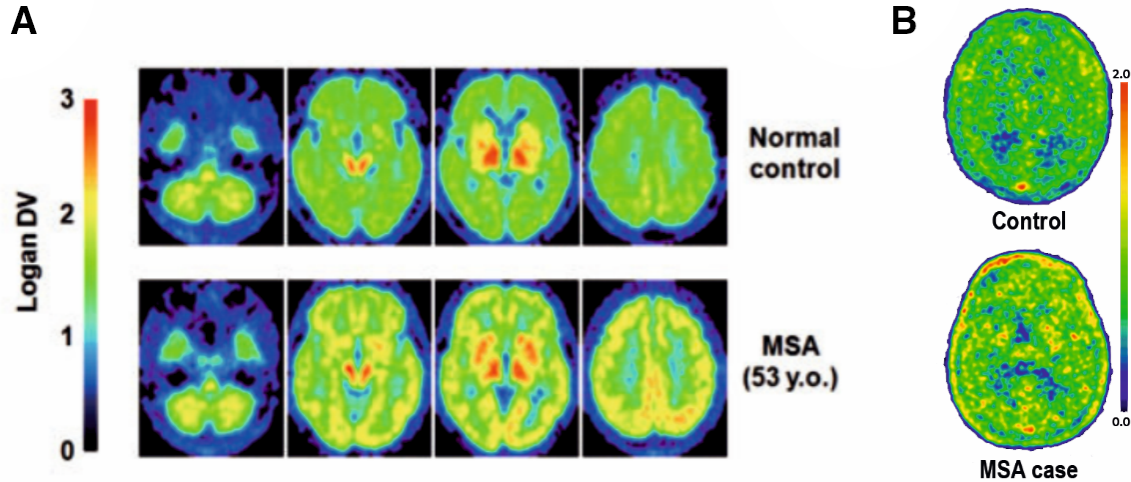
Attempts made to image Lewy pathology in vivo in multiple system atrophy (MSA). (**A**) PET images of a normal subject and a case of MSA imaged with [^11^C]BF227. The MSA case show increased cortical, basal ganglia, and white matter signal compared to the normal case, reflecting αSyn aggregates.^87^ (**B**) PET images of a healthy control case and an MSA case imaged with [^11^C]PBB3. The MSA brain showed mild uptake in basal ganglia, frontal and parietal, and cortexes compared to the healthy control brain, which showed a lack of regionally specific binding.^90^

Amyloid‐β (Aβ), tau and αSyn form similar β‐sheet structures on aggregation.^79^, ^80^, ^81^ Presumably, imaging probes binding to Aβ or tau aggregates have the potential to bind to αSyn aggregates. Hence, the radiotracers [^11^C]PIB, [^11^C]BF227, [^11^C]‐PBB3, and its structural analog [^3^H]‐C05‐01 were evaluated for imaging αSyn aggregates. [^11^C]PIB is a thioflavin‐T derivative and the gold standard for staining β‐sheet structured protein aggregates. Although PIB exhibited high binding affinity (K_d_ = 4 nM) for recombinant αSyn fibrils,^82^ it did not bind LBs‐containing DLB brain homogenates.^83^ In addition, PIB did not show interaction with PD brain sections containing LBs/LNs on autoradiography,^82^ and displayed poor binding selectivity for αSyn versus Aβ in DLB brain sections.^83^ [^18^F]BF227 was developed to image Aβ plaques in AD.^84^ The high binding affinity of [^18^F]BF227 (K_d_ = 9.6 nM) for recombinant αSyn fibrils conflicted with failed binding to LBs‐containing DLB brain homogenates^85^ and failed detection of GCIs in MSA brain with autoradiography.^86^ [^11^C]BF227 showed higher uptake in GCI‐rich brain regions of MSA patients relative to control subjects (Fig. 3A), however, the group differences were small, and many individual values overlapped indicating low in vivo selectivity for aggregated αSyn.^87^ In addition, as expected, this radiotracer showed high binding affinity to Aβ plaques in AD patients and clearly differentiated them from control subjects,^84^ demonstrating high in vivo affinity for Aβ plaques. [^11^C]‐PBB3 emerged as one of the first generation tau radiotracers.^88^ [^11^C]‐PBB3 showed in vitro autoradiographic binding to GCIs only in a subset of MSA cases,^89^ and in vivo higher uptake in the brain of one MSA case compared to control subject (Fig. 3B).^90^ These initial studies suggested that [^11^C]‐PBB3 displays some binding to aggregated αSyn and the PBB3 analog [^3^H]‐C05‐01 was developed and displayed a reasonable binding affinity (K_d_ = 24 nM) to recombinant αSyn fibrils. In vitro autoradiography using tissue microarrays and fresh‐frozen brain tissue showed that, whereas [^3^H]C05‐01 selectively binds to αSyn aggregates in PD and MSA brain, the ligand also selectively binds to Aβ and tau aggregates in AD, and therefore, has limited specificity for αSyn.^91^ Apparently, these repurposed radiotracers lack selectivity toward αSyn aggregates, and therefore, are of no interest for further development because of limited applicability in α‐synucleinopathies.

## Re‐Explored Chemical Entities

[^11^C]anle253b is an analog of anle138b, an αSyn fibrilization inhibitor with a demonstrated therapeutic activity in rodent models.^70^, ^92^ In vitro binding assays showed that [^11^C]anle253b preferentially binds to αSyn fibrils over oligomeric and monomeric species. However, the high log *P* of 5.21 and atypical brain uptake kinetic curves in healthy rats indicated the need for physicochemical properties optimization.^93^ More recently, MODAG‐001 was developed by modifying the chemical structure of anle253b to overcome the suboptimal in vivo pharmacokinetic properties of [^11^C]anle253b. In in vitro binding assays, [^3^H]MODAG‐001 combined a very high binding affinity toward recombinant αSyn (K_d_ = 0.6 ± 0.1 nM) with a good selectivity versus Aβ (K_d_ = 20 ± 10 nM) and tau (K_d_ = 19 ± 6.4 nM). [^11^C]MODAG‐001 showed suitable pharmacokinetic and biodistribution properties in mouse, and (d3)‐[^11^C]MODAG‐001 binding to recombinant αSyn fibrils was confirmed in fibril‐inoculated rat striata using in vivo PET imaging, but two radiometabolites from both radiotracers were detected in plasma and brain. Additionally, in vitro autoradiography showed no binding of (d3)‐[^11^C]MODAG‐001 to αSyn aggregates in human brain sections of DLB cases. Nonetheless, MODAG‐001 is still a promising lead structure for further development as it combines a high affinity and good selectivity with a suitable pharmacokinetics and biodistribution properties.^94^ [^11^C]14 ((3R)‐7‐([4′‐[11C]methoxynapthylen‐1‐yl]methyl)‐5‐oxo‐8‐(3‐(trifluoromethyl)‐phenyl)‐2,3‐dihydro‐5H‐thiazolo[3,2‐a]pyridine‐3‐carboxylic acid acetoxymethyl ester) is an analog of thiazolo‐2‐pyridone, FN075, an αSyn fibrillization accelerator, penetrated the brain in nonhuman primates, but showed poor pharmacokinetic properties for in vivo imaging with low initial brain uptake (≈0.8 standard uptake value), slow brain penetration (peak at 10 minutes post injection), and slow washout. Nearly half the radioactivity signal was still detectable as nonspecific binding by the end of the PET scan, which could be attributed to the high lipophilicity (cLogP = 6.1).^95^ The lead compound [^18^F]46a developed based on the 3‐(benzylidene)‐indolin‐2‐one scaffold showed good binding affinity toward αSyn (K_d_ = 8.9 nM) and selectivity over Aβ (K_d_ = 271 nM) and tau (K_d_ = 50 nM) in in vitro binding assays. However, the high log *P* of 4.18 and structural limitations indicated potentially high nonspecific binding that terminated further development.^96^

## In Silico Modelling and High‐Throughput Screening

This strategy uses chemical entities with reasonable binding properties to aggregated αSyn to generate potential ligands that undergo computational evaluation on αSyn fibrils structure to test ligands engagement with αSyn fibrils and feasibility for binding sites detection. The iodo‐derivative [^125^I]61 (2‐(3,4‐dimethylphenoxy)‐N‐(3‐(4‐[125I]iodophenyl)isoxazol‐5‐yl)acetamide) bound to confirmed binding site location on αSyn fibrils structure and showed high in vitro binding affinity to αSyn fibrils (K_d_ = 1.06 nM) extracted from the brain of PD mouse model and 5‐fold selectivity over Aβ_42_ fibrils (K_d_ = 5.56 nM). Using in vitro autoradiography, [^125^I]61 demonstrated binding to αSyn‐rich regions on sections from the same PD mouse model, but also nonspecific binding. The non‐ideal physicochemical properties of [^125^I]61 made this radiotracer unsuitable for further evaluation.^97^ [^18^F]2FBox showed high in vitro binding affinity toward αSyn fibrils (K_d_ = 3.3 ± 2.8 nM, B_max_ = 0.128 ± 0.025 pmol/nmol of fibril) and selectivity over Aβ_1−42_ (K_d_ = 145.3 ± 114.5 nM, B_max_ = 0.592 ± 0.251 pmol/nmol of fibril). However, [^18^F]2FBox failed to label LBs in PD and MSA brain sections. Additionally, [^18^F]2FBox non‐selectively detected both αSyn and Aβ_1−42_ fibrils in rats striatum injected with both proteins using in vitro autoradiography and failed to detect injected αSyn fibrils in rats despite the reasonable pharmacokinetic properties using in vivo PET.^98^

## Lessons Learned from αSyn Radiotracer Development

**FIG 4:**
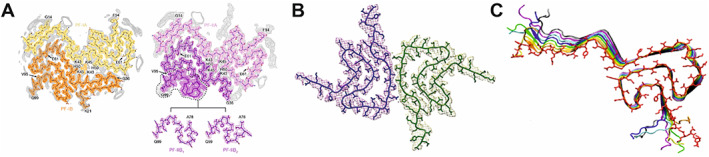
Two structural models of αSyn fibril extracted from multiple system atrophy (MSA) brain resolved by cryo‐EM^116^ (**A**) differ from structural models of recombinant human αSyn fibril resolved by cryo‐EM^115^ (**B**) and solid‐state NMR^112^ (**C**).

Collectively, the above mentioned small‐molecule ligands are not yet suitable for in vivo imaging of aggregated αSyn because of suboptimal binding affinity and/or specificity and physicochemical properties. However, many lessons have been learned that will help in the successful development of αSyn‐selective radiotracer in the future. First, the radiotracer should possess high binding affinity and selectivity; blood brain barrier (BBB) penetration; adequate initial brain uptake and washout; absence of BBB‐penetrating radioactive metabolites; absence of substrate activity on BBB efflux transporters, such as P‐glycoprotein; optimal physicochemical properties, such as lipophilicity (logD 1–3 at pH 7.4) for effective BBB penetration via passive diffusion and limited nonspecific tissue binding.^99^ Second, in vivo imaging of aggregated αSyn is challenging and the following aspects must be considered early in development. (1) Although αSyn aggregates (oligomers and fibrils) do spread in the brain with evidence for extracellular propagation,^37^, ^100^, ^101^, ^102^ they are predominantly present in intracellular inclusion bodies.^27^ Consequently, the radiotracer needs to cross the BBB and ideally penetrates the cell membrane either by an active transport mechanism or passively to bind intracellular αSyn aggregates. (2) Different types of cells are involved in different α‐synucleinopathies. In PD and DLB, αSyn accumulates in the neurons as LBs and LNs,^31^ whereas in MSA αSyn accumulates in oligodendrocytes as GCIs.^33^ Additionally, GCIs are structurally different from LBs and LNs^35^, ^36^ (also see point 6 below). An important implication of that to radiotracers design is that structural modifications could be necessary for imaging αSyn aggregates in oligodendrocytes GCIs and neurons LBs/LNs. (3) Unlike Aβ plaques but like tau, αSyn aggregates present in the brain are low in abundance and small in size, which is demanding for adequate in vivo detection and visualization. The area covered by LBs in DLB brain sections was found to be 40× smaller than that of Aβ plaques.^83^ Nonetheless, in vitro binding assays using recombinant αSyn fibrils and fibrils from PD brain showed that αSyn has sufficient binding site density to enable in vivo imaging, which is similar to tau, but less than Aβ plaques.^103^ Moreover, the binding site density of αSyn (SIL23, αSyn fibrils B_max_ = 108–895 nmol/L^103^) is significantly higher than that of pre‐ and post‐synaptic receptors in the dopaminergic and serotonergic systems (raclopride, D2 receptor B_max_ = 30–40 nmol/L), for which PET radiotracers have been developed.^104^ (4) The small‐molecule ligands developed till now bind to the αSyn β‐sheet structure, which is similar to tau NFTs and Aβ plaques.^79^, ^80^, ^81^ Additionally, αSyn co‐localizes with numerous other proteins inside the inclusion bodies including tau.^27^ Therefore, developed ligands suffered from poor binding selectivity. These facts are problematic because Lewy pathology in PD and DLB often co‐occurs with Aβ and tau pathologies as a result of co‐existing AD and/or other tauopathies^105^, ^106^ which has been shown by amyloid PET imaging.^107^ To overcome this challenge, more efforts are needed with the current and novel in silico approaches to develop small‐molecule ligands that are specific for aggregated αSyn binding‐sites.^97^, ^108^ (5) αSyn aggregates form soluble oligomers and insoluble fibrillary structures in α‐synucleinopathies.^109^, ^110^, ^111^ Ideally, these different structural conformations demand radiotracers with conformation‐specific binding affinities for accurate and selective in vivo detection. (6) The majority of used αSyn materials in radiotracer development are recombinant fibrils. αSyn fibrils generated in vitro are not reliable for the evaluation of αSyn ligands because of poor reproducibility, and more importantly, not representing aggregated αSyn in the human brain. Thus far, screening ligands for αSyn aggregates has been based on the available NMR^112^ and cryogenic electron microscopy (cryo‐EM)^113^, ^114^, ^115^ structures of recombinant αSyn fibrils (Fig. 4B,C). More recently, the cryo‐EM structure of authentic αSyn fibrils from the MSA brain has been solved for the first time, revealing that there are two types of αSyn fibrils that consist of four distinct protofibrils, which differ in conformation, folding, and predominance in different brain regions (Fig. 4A).^116^ These advancements are highly relevant to the development of αSyn‐specific radiotracer considering that compared to the MSA‐αSyn fibrils, in vitro‐made αSyn fibrils are smaller, differ in conformation, and consist of fewer protofibrils. Unlike αSyn fibrils from MSA brain, αSyn fibrils from DLB or PD brain are thinner and do not twist, which precluded solving their 3D cryo‐EM structure. However, based on 2D analysis, αSyn from MSA and DLB are distinct.^116^ Therefore, using human brain tissue from α‐synucleinopathies brains is the most reliable, although availability is problematic, especially without other co‐pathologies.^117^ (7) Suitable rodent models of Lewy pathology need to be used when evaluating the in vivo binding properties of αSyn radiotracers. For a reliable evaluation, rodent models must develop sufficient Lewy pathology inclusion bodies using αSyn materials from α‐synucleinopathies brain. Several reliable models exist, including the genetic models developed in mouse based on *SNCA* wild type or mutated variants overexpression, but the downside of the genetic models is that the developed Lewy pathology is generally mild and manifest after several months.^118^, ^119^, ^120^ A more practical alternative is the seeding model developed in mouse and rat by injecting αSyn aggregates in the brain, which can develop acute, abundant, and progressive Lewy pathology.^121^, ^122^ Those models develop αSyn aggregates with an onset of 1 month, which is not feasible with the genetic models.^123^ Viral vector models are another alternative, which are developed in mouse and rat using a virus vector to deliver *SNCA* to the substantia nigra dopaminergic neurons where αSyn is overexpressed. Those models develop αSyn aggregates with an onset of days to weeks. An additional advantage of the viral vector models is that αSyn is overexpressed only in the substantia nigra dopaminergic neurons; therefore, αSyn aggregates are only localized to the nigrostriatal pathway, which is substantia nigra and striatum. This characteristic could be beneficial for evaluating radiotracers in vivo binding selectivity. Ideally, a radiotracer selective for αSyn aggregates should demonstrate binding in the substantia nigra and the striatum because of the presence of αSyn aggregates, and no nonspecific binding in other brain regions because of the absence of αSyn aggregates.^124^, ^125^, ^126^ The double‐hit strategy combines the genetic and seeding models,^127^, ^128^, ^129^ or the seeding and viral vector models^130^, ^131^ to generate αSyn aggregates that are even more abundant than a single model could generate.

## Antibody‐Based Ligands: A Complementary Strategy for Imaging αSyn Aggregates

**FIG 5:**
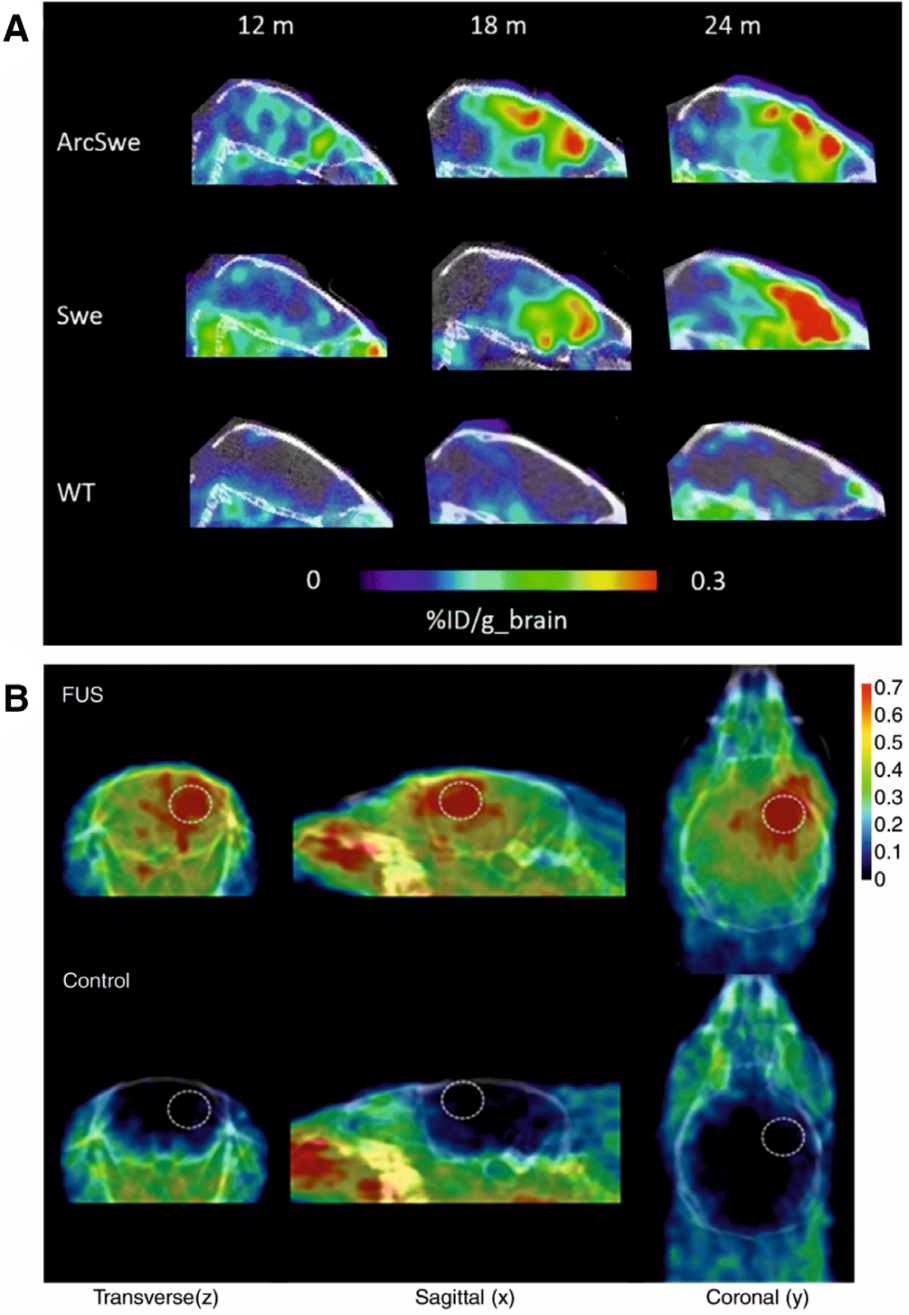
Strategies to overcome the limited passage of radiolabeled antibodies across the blood brain barrier. (**A**) Sagittal PET images obtained at 3 days after administration of the bispecific radioligand [^124^I]8D3‐F(ab′)_2_‐h158 in two mouse models of Alzheimer's disease (ArcSwe and Swe) and wild‐type mice at 12, 18, and 24 months.^149^ (**B**) Representative decay‐corrected PET/CT fused images in focused ultrasound (FUS)‐treated animals (top row) and control animals (bottom row) obtained 15 minutes after injection of ^68^Ga‐bevacizumab. Dashed circles, focused ultrasound targeting sites.^143^

Antibody‐based radiotracers offer superior binding affinity and specificity over small‐molecule radiotracers, and the capacity to target different structural conformations of a protein. Typically, antibodies have exceptionally high target binding affinity in the subnanomolar range,^132^ such affinity range for small molecules is unattainable. In the case of αSyn aggregates, antibodies binding affinity is reported in the picomolar range (BIIB054 K_d_ = ~120 pM,^60^ MEDI1341 K_d_ = 74 pM^63^), whereas the highest reported binding affinity of a radiolabeled small‐molecule is in the nanomolar range (MODAG‐001 K_d_ = 0.6 ± 0.1 nM.^94^ Radiolabeled antibodies demonstrated a proof of concept in imaging Aβ aggregates using AD rodent models (Fig. 5A).^133^, ^134^, ^135^, ^136^ Because antibodies are large molecules by nature, the primary limitation for radiolabeled antibodies is their restrained passage across the BBB. Approximately, only 0.1% of a peripherally administered antibody dose reaches the brain.^137^, ^138^ Active transport into the brain by receptor‐mediated transcytosis is one way to overcome this limitation where the transferrin receptor has been a successful shuttling system for enhanced targeting of Aβ aggregates by radiolabeled antibodies in AD mouse models.^133^, ^136^, ^139^, ^140^ Another way is to use microbubble‐mediated focused ultrasound (FUS), which is a non‐invasive imaging technique for enhanced drug delivery to the brain by reversibly opening the BBB.^141^, ^142^ Oncological applications using FUS in combination with radiolabeled‐antibody PET radiotracers showed that this technique significantly enhanced BBB penetration of bevacizumab (Fig. 5B)^143^ and ^18^F‐FBPA‐F^144^ in glioma‐bearing mouse and rat models, respectively.

A secondary limitation associated with radiolabeled antibodies is the typically slow pharmacokinetics of antibodies, that is slow target accumulation and clearance, which requires the use of long‐lived radionuclides for PET imaging and therefore, results in a high radiation exposure.^145^ This can partly be overcome by the introduction of total body PET/CT scanners, which have significantly higher sensitivity than the current standard PET/CT scanners.^146^ Another approach to overcome this limitation is the in vivo pretargeting. In this approach, a slow kinetic tagged‐antibody is administered and allowed to bind the desired target days before a fast kinetic radiolabeled agent with a short‐lived radionuclide is administered and both components having to bind to each other in the body^147^ and more recently in the brain.^148^ This way, the pretargeting strategy significantly reduces the radiation exposure as it allows the use of short‐lived radionuclides that would otherwise be incompatible with antibodies. Sehlin and colleagues^149^ thoroughly reviewed the antibody‐based ligands strategy and the associated limitations for imaging misfolded proteins in the brain, and Van Dongen and colleagues^150^ reviewed the emerging application of antibodies in a theranostic setting where the same antibody is used for diagnosis as well as therapy.

In the case of imaging aggregated αSyn with radiolabeled antibodies, the predominant intracellular localization of aggregated αSyn^27^ forms an intrinsic accessibility problem. Unlike passing the BBB, there does not seem to be an immediate solution for this limitation. Nonetheless, radiolabeled antibodies could still have a potential application in imaging the extracellular αSyn aggregates spreading in the brain, although it has to be seen whether extracellular αSyn concentrations are high enough to allow PET imaging. αSyn cell‐to‐cell spreading has been well demonstrated using in vitro cell culture studies^151^, ^152^ and animal models.^130^, ^153^ In humans, LBs and LN have been shown to spread from host to graft dopaminergic neurons in the substantia nigra of PD patients (Fig. 1B),^100^ which has been replicated in a PD mouse model.^101^ Additionally, Lewy pathology propagation throughout the central nervous system (CNS)^37^ strongly suggests αSyn spreading between interconnected brain regions. In silico modeling for the development of MEDI1341 antibody, which binds monomeric and aggregated αSyn, predicted that achieving target affinity of <100 pM is required to potently bind extracellular levels of αSyn in the brain. Accordingly, MEDI1341 (K_d_ = 74 pM) showed to successfully bind and lower the levels of extracellular αSyn in rats, monkeys, and mouse model of Lewy pathology.^63^ The case of MEDI1341 antibody demonstrates that an antibody can successfully bind extracellular αSyn aggregates if a similar affinity level is accomplished, even though experimental measurements of the needed level of extracellular αSyn aggregates in the brain for an antibody to bind successfully are lacking. Another potential application of radiolabeled antibodies in imaging aggregated αSyn is that antibodies could have higher binding specificity for particular target species compared to small molecules. It has been demonstrated in AD mouse models that a radiolabeled antibody binding to Aβ oligomers and protofibrils is better able to monitor changes in Aβ levels after therapeutic intervention compared to a small molecule tracer binding only to Aβ fibrillar structures.^154^ Considering that imaging intracellular αSyn aggregates using radiolabeled antibodies seems to be infeasible, it could be that a radiolabeled antibody binding predominantly to spreading αSyn oligomers, but not fibrils or vice versa, in the extracellular domain is better able to monitor changes in αSyn aggregates level and therapy response than a small‐molecule radiotracer binding predominantly to intracellular αSyn aggregates. Collectively, the evidence from the above‐mentioned studies justifies evaluating the potential use of radiolabeled antibodies for imaging extracellular αSyn aggregates.

## Future Perspective

Α‐synucleinopathies and PD in particular will continue to be a heavy burden on healthcare systems and societies with the increasing prevalence of these disorders. Clearly, there is a paramount diagnostic and therapeutic unmet medical need to be addressed in α‐synucleinopathies. The availability of the first αSyn PET or SPECT radiotracer will have a significant potential in resolving these unmet needs and improve our understanding of Lewy pathology and α‐synucleinopathies. An αSyn radiotracer could possibly achieve early and differential diagnosis of the different α‐synucleinopathies. Additionally, it could expedite the development of the several promising disease‐modifying agents currently in clinical trials. Although developing a radiotracer specific for αSyn aggregates proved to be challenging, our understanding of this complex task improved vastly and considerable progress has been made. The learned lessons from previously tested radiotracers must be carefully considered in future developments, where both small‐molecule ligands and antibodies should be the focus of development. For small‐molecule ligands, the in silico approach is particularly promising to overcome the specificity hurdle. Small‐molecule ligands offer superior BBB passage, cellular penetration, and target‐reachability compared to radiolabeled antibodies, which are limited by poor BBB passage and cellular penetration, but offer superior binding specificity and the potential for theranostic applications. Additionally, based on the current evidence from radiolabeled antibodies evaluated in preclinical applications in AD, and the localization of αSyn aggregates in the brain, radiolabeled antibodies could have a complementary rather than an alternative role to small‐molecule ligands. Neither radiolabeled antibodies nor small‐molecule ligands are currently capable of imaging the complete spectrum of αSyn aggregates in the brain. Small‐molecule ligands are better suited for imaging total (extra and intracellular) αSyn aggregates, whereas radiolabeled antibodies could be better suited for imaging extracellular αSyn aggregates and for monitoring the levels of αSyn aggregates and therapy response in α‐synucleinopathies.

## Author Roles

(1) Research project: A. Conception, B. Organization, C. Execution; (2) Statistical Analysis: A. Design, B. Execution, C. Review and Critique; (3) Manuscript: A. Writing of the First Draft, B. Review and Critique. O.M.A. defined the review scope and wrote the manuscript with an input from G,v.D., E.v.d.G., and W.B. O.M.A. and L.S. designed the literature search queries and performed the literature search. All co‐authors critically reviewed the manuscript.

## Financial Disclosures

None of the authors has financial disclosures during the past 12 months.

## Data Availability

The data that support the findings of this study are openly available in PubMed and Embase databases.
